# The Impact of Marijuana on Antidepressant Treatment in Adolescents: Clinical and Pharmacologic Considerations

**DOI:** 10.3390/jpm11070615

**Published:** 2021-06-29

**Authors:** Samuel E. Vaughn, Jeffrey R. Strawn, Ethan A. Poweleit, Mayur Sarangdhar, Laura B. Ramsey

**Affiliations:** 1 Division of Child and Adolescent Psychiatry, Department of Pediatrics, Cincinnati Children’s Hospital Medical Center, College of Medicine, University of Cincinnati, Cincinnati, OH 45229, USA; strawnjr@mail.uc.edu; 2 Department of Psychiatry and Behavioral Neuroscience, College of Medicine, University of Cincinnati, Cincinnati, OH 45219, USA; 3 Division of Clinical Pharmacology, Cincinnati Children’s Hospital Medical Center, College of Medicine, University of Cincinnati, Cincinnati, OH 45229, USA; laura.ramsey@cchmc.org; 4 Division of Biomedical Informatics, Department of Pediatrics, Cincinnati Children’s Hospital Medical Center, College of Medicine, University of Cincinnati, Cincinnati, OH 45229, USA; poweleen@mail.uc.edu (E.A.P.); mayur.Sarangdhar@cchmc.org (M.S.); 5 Division of Research in Patient Services, Cincinnati Children’s Hospital Medical Center, College of Medicine, University of Cincinnati, Cincinnati, OH 45229, USA; 6 Department of Biomedical Informatics, College of Medicine, University of Cincinnati, Cincinnati, OH 45219, USA; 7 Cancer and Blood Diseases Institute, Cincinnati Children’s Hospital Medical Center, Cincinnati, OH 45229, USA

**Keywords:** selective serotonin reuptake inhibitors, marijuana, CYP2C19, drug–drug interaction, adverse reactions

## Abstract

The neuropharmacology of marijuana, including its effects on selective serotonin reuptake inhibitor (SSRI)/antidepressant metabolism and the subsequent response and tolerability in youth, has received limited attention. We sought to (1) review clinically relevant pharmacokinetic (PK) and pharmacodynamic (PD) interactions between cannabinoids and selected SSRIs, (2) use PK models to examine the impact of cannabinoids on SSRI exposure (area under curve (AUC)) and maximum concentration (C_MAX_) in adolescents, and (3) examine the frequency of adverse events reported when SSRIs and cannabinoids are used concomitantly. Cannabinoid metabolism, interactions with SSRIs, impact on relevant PK/PD pathways and known drug–drug interactions were reviewed. Then, the impact of tetrahydrocannabinol (THC) and cannabidiol (CBD) on exposure (AUC_24_) and C_MAX_ for escitalopram and sertraline was modeled using pediatric PK data. Using data from the Food and Drug Administration Adverse Events Reporting System (FAERS), the relationship between CBD and CYP2C19-metabolized SSRIs and side effects was examined. Cannabis and CBD inhibit cytochrome activity, alter serotonergic transmission, and modulate SSRI response. In PK models, CBD and/or THC increases sertraline and es/citalopram concentrations in adolescents, and coadministration of CBD and CYP2C19-metabolized SSRIs increases the risk of cough, diarrhea, dizziness, and fatigue. Given the significant SSRI–cannabinoid interactions, clinicians should discuss THC and CBD use in youth prescribed SSRIs and be aware of the impact of initiating, stopping, or decreasing cannabinoid use as this may significantly affect es/citalopram and sertraline exposure.

## 1. Introduction

Anxiety and depressive disorders, the most common mental health conditions in children and adolescents [1], are frequently treated with selective serotonin reuptake inhibitors (SSRIs) [2,3,4]. However, treatment response varies considerably [5,6,7,8] and is often difficult to predict [9]. The considerable variation in SSRI response in adolescents with depressive and anxiety disorders is complex and is related to multiple factors, including comorbidity, pharmacogenetics, and substance use [10,11,12].

Youth with depression are twice as likely to report cannabis use and use amongst depressed teens has increased more rapidly over the past 15 years compared to their peers [13]. Further, an estimated one in three twelfth graders (17–18 years old) used marijuana in the past year and 6% of these adolescents used marijuana on a daily basis. Similarly, among eighth and tenth graders, daily use increased since 2018, with 1.3% of eighth graders (an 85.7% increase in 13–14-year-olds) and 4.8% of tenth graders (a 41.2% increase in 15–16-year-olds) reporting daily use (NIDA, NIH, NHHS).

Cannabinoids and several SSRIs are hepatically metabolized by CYP2C19 and CYP2D6. CYP2D6, and CYP2C19 metabolize many neuropsychiatric medications. CYP2D6 has over 100 known allelic variants and over 20 of these polymorphisms impact function. Broadly, these alleles are classified as normal function, decreased function, and no function [14]. As each individual patient inherits two alleles, phenotypes depend on the combination of inherited alleles. Four metabolic phenotypes have been predicted for CYP2D6: ultrarapid metabolizers inheriting duplications of functional alleles (1–20% of patients), normal (extensive) metabolizers inheriting at least one normal function allele (19–63%), intermediate metabolizers inheriting at least one decreased function or no function allele (14–72%), and poor metabolizers (0–6%) inheriting only no function alleles. Fluoxetine, fluvoxamine, and paroxetine are metabolized by CYP2D6 [15]. 

**Figure 1:**
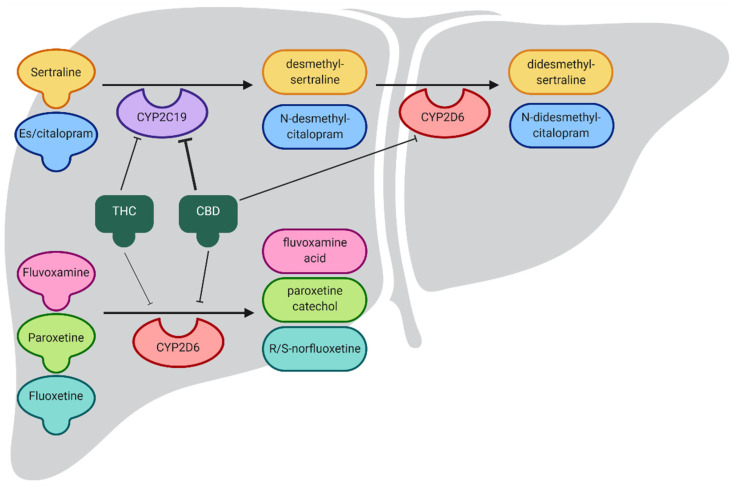
SSRI metabolism and the effects of cannabidiol (CBD) and tetrahydrocannabinol (THC) on CYP2C19 and CYP2D6 activity. Both CBD and THC inhibit CYP2C19, decreasing the metabolism of CYP2C19 substrates, including sertraline and es/citalopram, delaying production of active metabolites. THC and CBD have a reduced effect on CYP2D6, indicated by the thickness of the lines showing inhibition.

CYP2C19 is highly polymorphic, with over 30 identified allelic variants. These can be grouped in broad functional groups. There are five predicted phenotypes for CYP2C19: ultrarapid metabolizers carrying two increased function alleles (0–5% of patients), rapid metabolizers carrying an increased function allele paired with a normal function allele (1–27% of patients), normal (extensive) metabolizers carrying two normal function alleles (9–47%), intermediate metabolizers carrying one normal or increased function allele together with one decreased or no function allele (24–47%), and poor metabolizers carrying two decreased or no function alleles (2–46%). Citalopram, escitalopram, and sertraline are metabolized primarily by CYP2C19 [14,15,16] (Figure 1).

The predicted phenotypes are the focus of functional studies, which evaluate the impact of allelic variation on SSRI pharmacokinetics and clinical outcomes, including efficacy and tolerability. For example, we previously demonstrated that CYP2C19 metabolizer status impacts the outcomes with escitalopram/citalopram in youth with anxiety and depressive disorders [17,18]. Slower metabolizers were more likely to discontinue treatment compared to normal metabolizers, and were more likely to experience significant side effects, including weight gain and activation. 

For citalopram and escitalopram, the Clinical Pharmacogenetics Implementation Consortium (CPIC) recommends that clinicians treating CYP2C19 rapid and ultrarapid metabolizers consider alternative medications that are not significantly metabolized by CYP2C19 [15]. For sertraline, the difference in CYP2C19 ultrarapid metabolizers appears to be less pronounced, so no dose adjustment is recommended [15]. In CYP2C19 poor metabolizers, CPIC recommends a dose reduction of 50% for citalopram, escitalopram, and sertraline because elevated medication concentrations have been observed (e.g., citalopram/escitalopram) or more side effects have been reported (e.g., sertraline). These recommendations are based on data from adults, but pediatric data are increasingly reported [17,19,20,21] and will be assessed in the update of the CPIC SSRI guideline, expected to be published in late 2021 or early 2022. 

Variation in SSRI response may also relate to differences in SSRI exposure (e.g., blood concentration) among adolescents [12,22]. Importantly, marijuana use may also influence the pharmacology of SSRIs, including their pharmacokinetic profile. The pharmacology of cannabinoids may accentuate this variation in response in adolescents with these disorders. However, the neuropharmacology of marijuana use; interactions between marijuana and serotonergic transmission; and the effects of marijuana on an SSRI metabolism, response, and tolerability have received limited attention in the current literature. With this in mind, we sought to (1) describe the impact of marijuana on SSRI treatment in children and adolescents, (2) characterize and examine the effect of marijuana on enzymes that metabolize SSRIs with regard to variation in SSRI response in adolescents, and (3) examine the frequency of adverse events reported when SSRIs and cannabinoids are used concomitantly. Hereafter, we focus primarily on es/citalopram and sertraline as (1) these SSRIs are primarily metabolized by CYP2C19, (2) CPIC has issued genotype-specific dosing guidelines for these medications, and (3) CBD appears to have a greater interaction with CYP2C19 than CYP2D6.

## 2. Pharmacokinetic Interactions

### 2.1. Cannabis and Cannabidiol Impact Cytochrome Activity

Although cannabis contains hundreds of individual chemicals, we focus on cannabinoids in this review. The most abundant phytocannabinoids in cannabis are Δ9-tetrahydrocannabinol (THC) [23] and CBD [24,25]. Cannabinoids are metabolized in the liver predominantly by CYP2C9 and CYP3A4 [26], although CYP2D6 and CYP2C19 are also involved [27]. In addition to being metabolized by these enzymes, THC and CBD inhibit CYP2D6 [28] (Food and Drug Administration (FDA) Epidiolex, FDA news alert) and CYP2C19 [29] (FDA Epidiolex, FDA news alert, Sativex regulatory) in vitro (Table 1), as summarized in Zendulka, Dovrtelova [30]. Based on in vitro experiments and the estimated plasma drug levels, CBD was predicted to induce strong drug interactions with CYP2C19 and moderate interactions with CYP2D6 [31]. However, THC, taken orally or inhaled, was not predicted to induce drug interactions with CYP2C19 or CYP2D6 [31]. CBD also inhibits CYP2C19 in vivo [32,33,34]. Taken together, these data suggest that medications with a CYP2C19-dependent metabolism are likely to be susceptible to drug–drug interactions with cannabinoids [35].

### 2.2. Cannabis and Cannabidiol Potentially Interact with SSRIs

To date, no studies have examined the impact of marijuana use on SSRI efficacy or tolerability in adolescents. However, extrapolating from in vivo studies of the impact of cannabidiols on medications metabolized in a manner similar to SSRIs can provide guidance. Clobazam, which is metabolized primarily by CYP3A4 and CYP2C19, is slowly metabolized in CYP2C19 poor metabolizers, leading to accumulation of the active metabolite n-clobazam and increased side effects [36]. Coadministering clobazam and CBD increases plasma clobazam concentrations by about 60% and increases n-clobazam concentrations five-fold [37]. Similarly, CBD also increases the concentrations of stiripentol, another antiepileptic medication metabolized by CYP2C19 [38].

### 2.3. Pharmacokinetics of Cannabinoids

One significant challenge to studying the drug–drug interaction between marijuana and SSRIs is the difficulty in estimating marijuana exposure. As marijuana is composed of numerous cannabinoids, which vary in their concentration, including cannabis strain differences, isolation of specific cannabinoids, such as those found in over-the-counter products such as CBD oil [39], to medicinal preparations of synthetic cannabinoids, such as dronabinol. Of relevance to pharmacokinetic effects of cannabinoids and SSRIs, the method of preparation and the administration route significantly impact cannabinoid pharmacokinetics, as do frequency and the amount of use (summarized in Grotenhermen [40]). For example, oral THC bioavailability ranges from 5–20% and its absorption is delayed by 1–3 h as it is slowly absorbed by the gastrointestinal tract. Further, oral THC absorption is also influenced by sex, weight, and the presence of food [41]. By contrast, the pharmacokinetics of inhaled THC are more variable, with up to 50% of inhaled smoke exhaled and some localized pulmonary metabolism. This results in a bioavailability of 10–25%, faster absorption (generally within minutes) [42], and inhaled THC exhibits first-order kinetics [43].

### 2.4. Preliminary Models Of SSRI–Cannabinoid Pharmacokinetic Interactions

**Figure 2:**
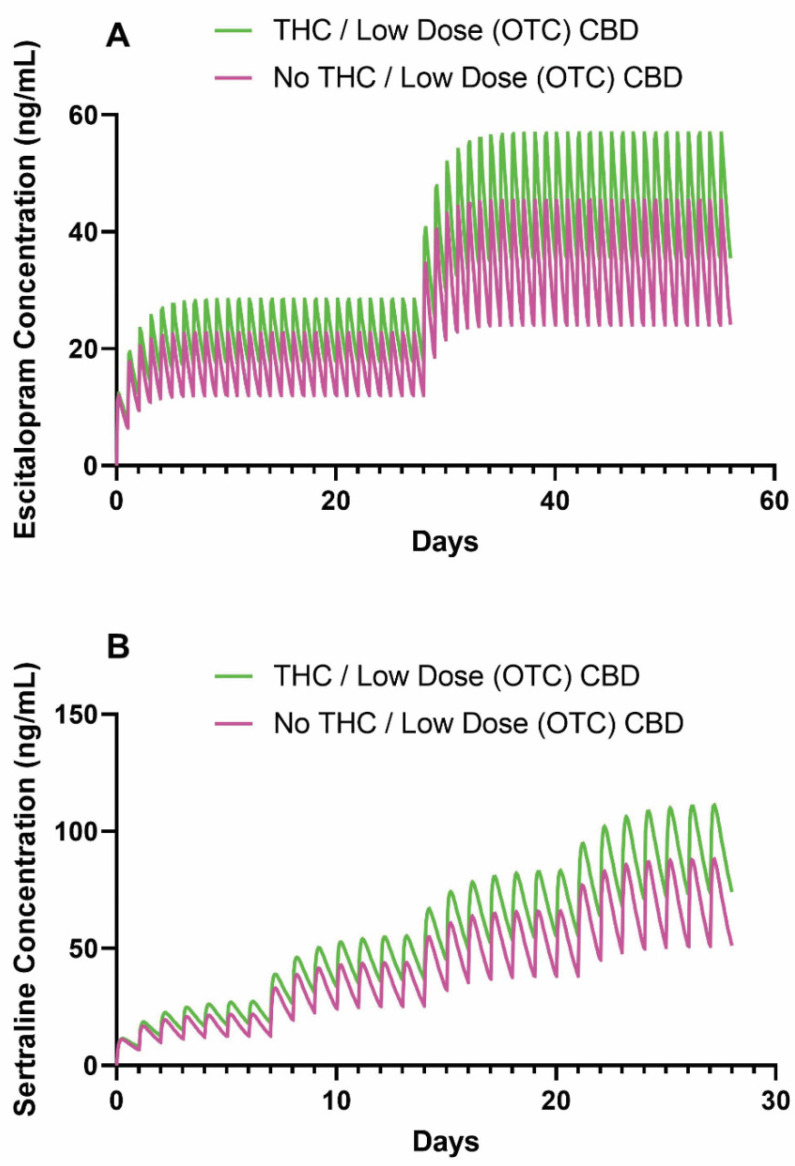
Simulated time course of (**A**) escitalopram and (**B**) sertraline plasma concentrations in adolescent CYP2C19 normal metabolizers consuming THC or low-dose CBD versus not consuming. For escitalopram, treatment was initiated at 10 mg daily and increased to 20 mg daily at week 4. For sertraline, treatment was initiated at 50 mg daily and increased by 50 mg each subsequent week until reaching 200 mg daily. Concurrent THC or low-dose CBD (5–15 mg/day) use with escitalopram or sertraline was simulated with the total body clearance reduced by 25%. Abbreviations: THC, tetrahydrocannabinol; CBD, cannabidiol.

We determined that the total body clearance of SSRIs may be reduced based on a recently reported pharmacokinetic interaction risk between oral THC or low-dose CBD and cytochrome enzymes [31]. Therefore, using previously estimated pharmacokinetic parameters and standard dosing regimens for an adolescent CYP2C19 normal metabolizer [12], we estimated the influence of concurrent THC or low-dose CBD (5–15 mg/day) use with escitalopram or sertraline (Figure 2).

In the pharmacokinetic model, the half-life of escitalopram with concurrent THC or low-dose over-the-counter CBD increased from 21.3 to 28.3 h. For escitalopram-treated adolescents receiving 20 mg/day, concurrent THC or low-dose over-the-counter CBD use increased the AUC and C_MAX_ by 35% (34.1 vs. 46.1 days ng/mL) and 25% (45.3 vs. 56.9 ng/mL), respectively (Table 2). Similarly, in the pharmacokinetic model for sertraline, concurrent THC or low-dose over-the-counter CBD increased the half-life of sertraline from 22.1 to 29.5 h. For sertraline-treated adolescents receiving 150 mg/day, concurrent THC or low-dose over-the-counter CBD use increased the AUC and C_MAX_ by 33% (54.2 vs. 72.1 days ng/mL) and 26% (66.0 vs. 83.2 ng/mL), respectively (Table 3). For both models, concurrent THC or low-dose CBD increased the time required for a patient to achieve a steady state.

We estimated the effects of concurrent THC or low-dose CBD use with sertraline or escitalopram using MwPharm (version 3.82, Mediware, Czech Republic [44]). MwPharm is a pharmacokinetic modeling program that enables users to approximate a patient’s clearance, volume of distribution, exposure, and concentration of individual medications (e.g., sertraline and escitalopram) based on previously published parameters. Model parameters of a medication are entered into the program, in addition to patient characteristics, including age, body size, sex, and medication/dosing history. Considering patient and medication information, the program simulates a time course of medication plasma concentrations for a patient, in addition to their estimated effects. For the escitalopram model, total body clearance was 25 L/h/70 kg, V1 was 12 L/kg lean body mass, and Ka was 0.8 h^−1^. For the sertraline model, total body clearance was 152 L/h/70 kg, V1 was 76 L/kg lean body mass, and Ka was 0.8 h^−1^. The effect of THC or low-dose CBD use was estimated based on recent clinical data [31] suggesting a reduction in escitalopram or sertraline clearance by 25% (to 18.75 and 114 L/h/70 kg, respectively). The area under the concentration–time curve (AUC) and the maximal concentration (C_MAX_) [45] were estimated from the final dose of each titration during steady state. Additional details of the pharmacokinetic modeling approach and specific pharmacokinetic parameters have been described (Strawn, Poweleit [12]).

### 2.5. Case Illustration of SSRI-Cannabinoid Pharmacokinetic Interactions

To illustrate the potential clinical impact of concurrent SSRI and cannabinoid use, we present the following. 

**Figure 3:**
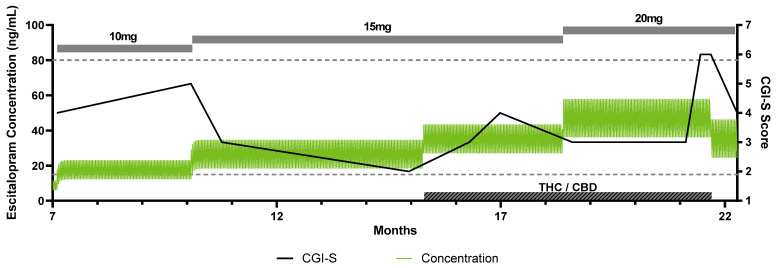
Time course with simulation of escitalopram plasma concentrations in adolescent CYP2C19 normal metabolizer taking low-dose over-the-counter CBD. This patient was being treated with escitalopram, treatment was well-controlled at 10 mg daily and increased to 15 mg daily at month 10 with worsening anxiety. The dose was increased to 20 mg daily at 18.5 months when anxiety again worsened. The green line indicates the simulated escitalopram plasma concentration plotted on the left y-axis. The black line indicates the CGI-S score, plotted on the right y-axis. The gray boxes indicate the escitalopram daily dose. The hashed gray box indicates when the patient was using the THC/CBD. The dashed lines indicate the upper and lower therapeutic reference range (15–80 ng/mL) in adults [46]. Abbreviations: CBD, cannabidiol; OTC, over-the-counter, CGI-S, Clinical Global Impression Scale-Severity of Illness, THC, tetrahydrocannabinol.

A 15-year-old adolescent who met the DSM-5 criteria for generalized anxiety disorder with panic attacks and recurrent, moderate major depressive disorder was treated with escitalopram 5 mg beginning from the age of 15 years 8 months. She had no relevant past medical history, no history of trauma, and was not treated with any other CYP2C19-metabolized medications. Her depressive and anxiety symptoms were in remission while treated with escitalopram 10 mg each morning for 3 months, and titrated up to 15 mg/day when anxiety worsened, including increasing panic attacks. Unbeknownst to her clinician or parents, she began consuming CBD/THC in edible form. As CBD/THC use continued, her anxiety symptoms worsened, her panic attacks became more intense and more frequent, and her depressive symptoms intensified. Escitalopram was titrated to 20 mg each morning, with some initial improvement, but her anxiety symptoms recrudesced. Following disclosure of the CBD/THC use to her parents and clinician, CBD/THC use stopped. Nausea, abdominal pain, and headaches were also reported at this time. A time course of the patient’s treatment, clinical outcome scores, and simulated escitalopram levels is illustrated in Figure 3. Using the same escitalopram plasma drug level simulation strategy as described above and shown in Figure 2, we simulated the change in escitalopram plasma concentrations in this patient with concurrent CBD/THC use. The side effects reported at the first visit following disclosure of CBD/THC use may relate to the modeled elevation in escitalopram exposure.

### 2.6. Real-World Cannabinoid and SSRI Interactions

The increased use of cannabinoids both recreationally and in clinical practice leads to a greater chance for coadministration with SSRIs and possible adverse events. Foster et al. [47] examined the U.S. Food and Drug Administration (FDA)’s Adverse Event Reporting System (FAERS) and found a significant number of adverse event reports involving marijuana or a derivative thereof, suggesting that there is a risk of marijuana–drug interaction. Reports have increased over time compared to other drug–supplement interactions, possibly correlating with the increased availability of marijuana-derived products, including both prescription and OTC THC- and CBD-derived medical and recreational products. Data on direct clinical interactions are slowly emerging as evidenced by a recent case report of a cannabis hyperemesis syndrome that may have occurred at least in part due to concurrent SSRI use [48].

### 2.7. Data Mining of Large-Scale Clinical Effects and Hypothesis Generation Using U.S. Food and Drug Administration’s Adverse Event Reporting System (FAERS) Data 

Adverse events data reported to FAERS and normalized within AERSMine [49] were used to identify differential rates of side effects typically associated with antidepressants. We analyzed ~15 million patient reports from the FDA for use of CYPC219-metabolized medications (sertraline, escitalopram, and citalopram, *n* = 427,932), cannabinoids (tetrahydrocannabinol, cannabidiol, and cannabinoids, *n* = 7008), and their combination (*n* = 421). The three mutually exclusive cohorts were analyzed for their frequencies of side effects. Side-effect frequencies in the CYP2C19 medication cohort were used as a baseline for comparison to the cannabinoid and combination cohorts. Drug label data were extracted from Lexicomp [50] and 23 side effects with >5% frequency on the sertraline or escitalopram label were used for benchmarking our comparative analysis. Standard pharmacovigilance metrics, relative risks, and safety signals [51,52], were used to identify drug and drug combination side effect associations. The Benjamini, Hochberg, and Yekutieli test [53] was used for false discovery rate (FDR) correction using a significance threshold of 0.05.

### 2.8. Combination Of CYP2C19-Metabolized Medications and Cannabinoids Presents Elevated Risk of Antidepressant-Related Side Effects

**Figure 4:**
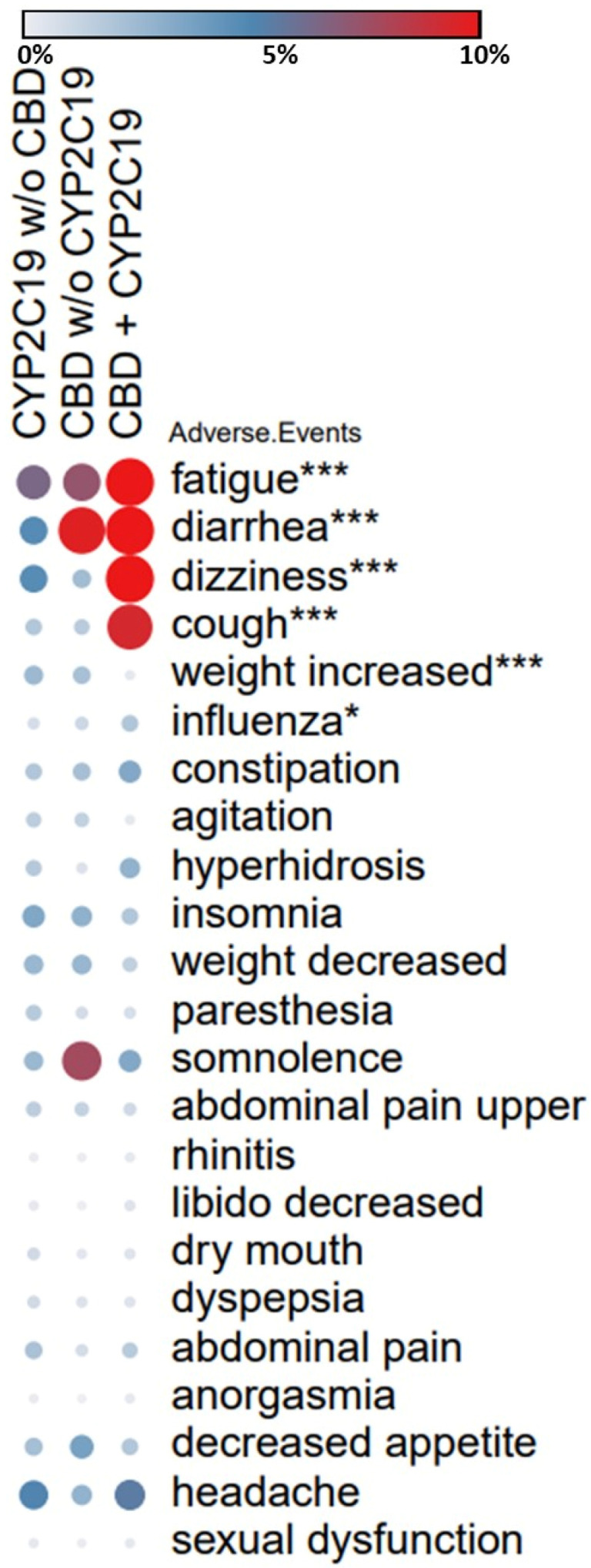
FAERS analysis of side effects in patients on antidepressants. The heatmap shows differential rates of side effects in patients on CYP2C19-metabolized medications (sertraline, es/citalopram), cannabinoids (CBD and THC) and the patients taking both as reported to FAERS. Rates of 23 side effects that are on the sertraline or es/citalopram label at >5% frequency were compared across three patient groups. Side effects with higher frequencies in the combination compared to the baseline (CYP2C19 medications without cannabinoids) and passing the FDR correction are highlighted using asterisks (* *p* < 0.05, *** *p* < 0.005). Frequencies of the side effects in the FAERS data are color coded as gray (0%), blue (5%), and red (10% or greater). Number of patients in each group: CYP2C19 (427,932), CBD (7008), CBD + CYP2C19 (421). Groups were mutually exclusive. Bejamini, Hochberg, and Yekutieli test used for FDR correction, significance threshold 0.05. FDR: false discovery rate.

To understand the relative safety profile of sertraline and es/citalopram in combination with cannabinoids, we analyzed 23 side effects with >5% frequency on sertraline or es/citalopram labels. Comparative analysis of these 23 side effects in the FAERS data revealed that patients on a combination of CYP2C19-metabolized medications and cannabinoids showed a disproportionately higher risk of certain side effects. Compared to patients taking sertraline or es/citalopram alone, patients in the combination group presented a 4.97-fold increase in cough, 3.33-fold higher rate of diarrhea, 3.29-fold higher rate of fatigue, 2.87-fold increase in dizziness, and 2.54-fold increase in influenza (FDR correction at 0.05, Figure 4). Conversely, weight gain was not common in patients in the combination group, 0.24% vs. 2.53% in the sertraline or es/citalopram group (*p* < 0.005, Figure 4, See Supplementary Table S1 for side effect frequencies in the FAERS data).

## 3. Pharmacodynamic Interactions

In addition to the potential impact of cannabinoids on SSRI pharmacokinetics, studies also examined the impact of cannabinoids on serotonin pharmacodynamics. In preclinical studies, mice administered a CB1 antagonist together with an SSRI showed improved performance on a behavioral test compared to either agent alone, and at lower doses [54]. In another pre-clinical study, administration of citalopram together with a CB1 antagonist increased 5-HT release in both the prefrontal cortex and locus cereulus of awake rats [55]. However, neither study examined the impact of concurrent medications on cytochrome metabolism, so it is unclear if these results are due to direct action at CB1 or changes in medication exposure due to hepatic drug–drug interactions.

**Figure 5:**
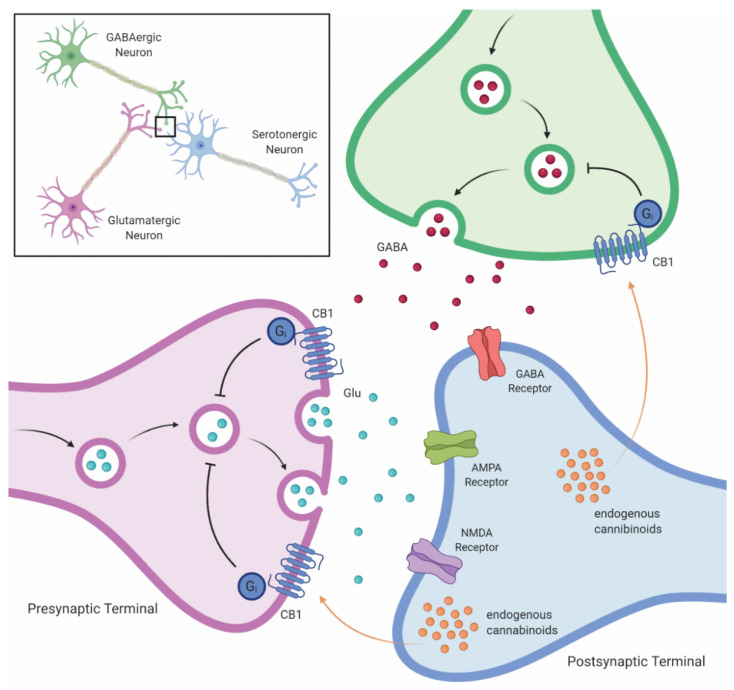
Cannabinoid-mediated impact on monoamine signaling in the brain. Cannabinoid-mediated stimulation of CB1 receptors on monoaminergic neurons leads to delayed transport of monoamines to the synapse, decreasing monoamines in the synapse. Abbreviations: GABA, gamma aminobutyric acid; Glu, glutamate; AMPA, α-amino-3-hydroxy-5-methyl-4-isoxazolepropionic acid; NMDA, N-methyl-D-aspartate; CB1, cannabinoid-1 receptor.

There is also a direct link between 5-HT signaling and the endocannabinoid system (Figure 5). Long-term cannabinoid administration alters the 5-HT receptor signaling, upregulating 5-HT2A activity and down-regulating 5-HT1A activity [56]. In addition, CB agonists upregulate the 5-HT2A receptor signaling [57]. This interaction appears to be bidirectional; serotonin-mediated 5-HT2 receptor activation increases endocannabinoid release and CB1 receptor activation [58]. Regarding exogenous cannabinoids, THC is an agonist at CB1 receptors, which may result in increased appetite, decreased working memory, and the euphoria associated with intoxication, whereas THC also agonizes CB2 [59] and 5-HT3, potentially conferring antiemetic properties (summarized in Pertwee [60]).

## 4. Discussion

Marijuana use is common in adolescents and its recreational and medicinal use has increased contemporaneously. The FDA has approved one cannabis-derived drug and three FDA-approved synthetic cannabis-related drugs. Further, OTC CBD products are increasingly marketed as treatments for anxiety and depression. As SSRIs are frequently prescribed in adolescents [61], we must better understand potential SSRI–cannabinoid interactions. Although there are limited studies of direct SSRI–cannabinoid interactions, accumulating data suggest the potential for PK and PD interactions.

As THC and CBD inhibit CYP2C19, and to a lesser degree CYP2D6, SSRIs metabolized by CYP2C19, including sertraline and es/citalopram, have a high likelihood of drug–drug interactions. This would be most likely observed in individuals with a poor metabolizer phenotype, who would already be likely to have increased drug concentrations (compared to normal metabolizers).

With the utility of cannabinoids as antiepileptic drugs, including recent FDA approval of the first cannabinoid-derived drug, Epidiolex^®^ (cannabidiol), drug–drug interactions between cannabinoids and other antiepileptic drugs metabolized by cytochrome enzymes have been described. Drug–drug interactions between cannabinoids and two substrates of CYP2C19, stiripentol and clobazam, have been described, supporting the possibility that cannabinoids affect other CYP2C19 substrates, including sertraline and es/citalopram [62]. Herein, we focused on SSRIs metabolized primarily by CYP2C19. Thus, our conclusions may not generalize across SSRIs. The increased side effects from concurrent cannabinoid and SSRIs we identified were all amongst the most common identified in the FDA package insert, and may relate to SSRI dose or plasma concentrations [63].

Our model predicts a clinically significant increase in plasma drug concentration variation with concurrent use of marijuana or low-dose CBD and SSRIs. This increase in plasma concentration would be magnified if high-dose CBD and SSRIs were concomitantly prescribed. As increased SSRI plasma concentration is associated with increased activation [64], the implications include a decrease in tolerability secondary to activation. Because medication-related side effects and clinical symptoms of disease can overlap and monitoring SSRI plasma drug levels is uncommon in clinical practice, the correlation between drug levels and side effects is poorly understood. Multiple trials found that common SSRI side effects increase as the SSRI dose is increased [63]. However, as the psychological effects of cannabinoids include euphoria and relaxation [65], activation may be masked with active concurrent use. Interestingly, given the prolonged half-life of cannabinoids, cannabis cessation, especially in the short term, may lead to worsened symptoms, potentially unmasking any underlying activation as cannabis-induced pharmacokinetic changes are likely to linger compared to the immediate psychological effects, which will return to baseline relatively quickly. Alternatively, as SSRI plasma concentration decreases following cannabis cessation, efficacy would also likely decrease. Considering the delayed release of 5-HT following CB1 stimulation, serotonergic pharmacodynamics are likely to be disrupted, decreasing drug efficacy.

Although the existing literature suggests a direct interaction between cannabinoids and SSRIs, direct studies are lacking. Studies examining SSRIs coadministered with cannabinoids (either CBD or recreational marijuana) and tolerability/efficacy are needed. In addition, it will be vital to measure SSRI plasma concentrations to better characterize the potential drug–drug interactions. 

## 5. Conclusions

Accumulating data suggest that CBD and THC affect concentrations of CYP2C19-metabolized SSRIs, including es/citalopram and sertraline. Using cannabidiol and or THC likely increases sertraline and es/citalopram concentrations in adolescents and may increase the risk of concentration-related SSRI side effects. Clinicians should consider inquiring as to the frequency and amount of THC and CBD use. Further, in sertraline- or es/citalopram-treated patients, stopping or decreasing marijuana or CBD use may decrease concentrations of sertraline or es/citalopram in stably treated patients.

## Figures and Tables

**Table 1:** Impact following tetrahydrocannabinol/cannabidiol administration on in vitro CYP enzyme activity [30].

	IC_50µ_ (µM) ^1^	
	CBD	THC
CYP2C19	0.30 ± 0.03	0.57 ± 0.22
CYP2D6	0.95 ± 0.50	1.28 ± 0.25
CYP3A4	0.38 ± 0.11	1.30 ± 0.34

^1^ CBD, cannabidiol; THC, tetrahydrocannabinol; IC_50µ_, binding-corrected 50% inhibitory concentration.

**Table 2:** Pharmacokinetic parameters at steady state for a model escitalopram normal metabolizer with concurrent cannabinoid use assuming a 25% reduction in clearance based on Bansal et al. [31].

	THC or Low-Dose CBD ^1^	
	−	+
*t*_1/2_ (h)	21.3	28.3
AUC_24_, 10 mg *q.d.* (days ng/mL)	17.1	23.1
C_max_, 10 mg *q.d.* (ng/mL)	22.7	28.4
AUC_24_, 20 mg *q.d.* (days ng/mL)	34.1	46.1
C_max_, 20 mg *q.d.* (ng/mL)	45.4	56.9

^1^ Abbreviations: *t*_1/2_, half-life; AUC_24_, area under the curve (24-h); *q.d.,* quaque die (once daily); THC, tetrahydrocannabinol; CBD, cannabidiol.

**Table 3:** Pharmacokinetic parameters at steady state for a model sertraline normal metabolizer with concurrent cannabinoid use assuming a 25% reduction in clearance.

	THC or Low-Dose CBD	
	−	+
*t*_1/2_ (h)	22.1	29.5
AUC_24_, 50 mg *q.d.* (days ng/mL)	18.0	23.7
C_max_, 50 mg *q.d.* (ng/mL)	21.9	27.4
AUC_24_, 100 mg *q.d.* (days ng/mL)	36.1	47.9
C_max_, 100 mg *q.d.* (ng/mL)	44.0	55.3
AUC_24_, 150 mg *q.d.* (days ng/mL)	54.2	72.1
C_max_, 150 mg *q.d.* (ng/mL)	66.0	83.2
AUC_24_, 200mg *q.d.* (days ng/mL)	72.3	98.45
C_max_, 200 mg *q.d.* (ng/mL)	88.0	111.7

## Data Availability

Adverse event data were obtained from the FDA Adverse Event Reporting System (https://fis.fda.gov/sense/app/d10be6bb-494e-4cd2-82e4-0135608ddc13/sheet/7a47a261-d58b-4203-a8aa-6d3021737452/state/analysis accessed on 14 March 2021).

## Supplementary Materials

The following are available online at https://www.mdpi.com/article/10.3390/jpm11070615/s1, Table S1: Side effect frequencies in FAERS data.
