# Supplementary material for: The Impact of Marijuana on Antidepressant Treatment in Adolescents: Clinical and Pharmacologic Considerations
Source: J Pers Med. 2021 Jun 29;11(7):615. doi: 10.3390/jpm11070615 (PMC8307883; doi:10.3390/jpm11070615)
Supplement: Supplementary file 1 [file jpm-11-00615-s001.zip › jpm-1268993-supplementary.pdf]

Adverse.Events	Sertraline_Frequency	Sertraline_label
Drug Label	Sertraline_Frequency	Sertraline_label
Unique.Patients		
fatigue***	>10% Frequency	1
diarrhoea***	>10% Frequency	1
dizziness***	>10% Frequency	1
cough***	0	0
headache	0	0
somnolence	>10% Frequency	1
constipation	5% to 9% Frequency	1
hyperhidrosis	5% to 9% Frequency	1
insomnia	>10% Frequency	1
decreased appetite	5% to 9% Frequency	1
influenza*	0	0
abdominal pain	5% to 9% Frequency	1
weight decreased	5% to 9% Frequency	1
abdominal pain upper	5% to 9% Frequency	1
paraesthesia	0	0
libido decreased	5% to 9% Frequency	1
dry mouth	0	0
dyspepsia	5% to 9% Frequency	1
anorgasmia	0	0
agitation	5% to 9% Frequency	1
sexual dysfunction	1% to 4% frequency	1
weight increased***	5% to 9% Frequency	1
rhinitis	0	0

Escitalopram_frequency	Escitalopram_label	Absolute Counts
**Escitalopram_frequency**	**Escitalopram_label**	**CYP2C19 w/o CBD**
		**427,932**
5% to 9% Frequency	1	27,457
>10% Frequency	1	20,421
5% to 9% Frequency	1	20,213
5% to 9% Frequency	1	7,978
>10% Frequency	1	21,727
>10% Frequency	1	10,977
5% to 9% Frequency	1	8,115
0	0	7,439
>10% Frequency	1	14,603
1% to 4% frequency	1	9,647
5% to 9% Frequency	1	3,196
1% to 4% frequency	1	9,081
0	0	11,273
1% to 4% frequency	1	6,533
1% to 4% frequency	1	7,218
5% to 9% Frequency	1	1,073
5% to 9% Frequency	1	3,874
5% to 9% Frequency	1	3,842
5% to 9% Frequency	1	532
0	0	6,572
>10% Frequency	1	992
0	0	10,830
5% to 9% Frequency	1	395

Absolute Counts CBD w/o CYP2C19	Absolute Counts CBD + CYP2C19		CYP2C19 w/o CBD Freq.CYP2C19 w/o CBD	CBD w/o CYP2C19 Freq.CBD w/o CYP2C19
**7,008**	**421**		**427,932**	**7,008**
509	89		6.42	7.26
676	67		4.77	9.65
168	57		4.72	2.40
114	39		1.86	1.63
200	23		5.08	2.85
540	14		2.57	7.71
155	14		1.90	2.21
38	12		1.74	0.54
204	8		3.41	2.91
260	8		2.25	3.71
76	8		0.75	1.08
58	7		2.12	0.83
188	6		2.63	2.68
92	4		1.53	1.31
57	3		1.69	0.81
3	2		0.25	0.04
23	2		0.91	0.33
38	2		0.90	0.54
	1		0.12	0.00
96	1		1.54	1.37
4	1		0.23	0.06
152	1		2.53	2.17
4	1		0.09	0.06

CBD + CYP2C19		FDR p
Freq.CBD + CYP2C19		FDR p
421	Fold-change	
21.14	3.29	< 0.005
15.91	3.33	< 0.005
13.54	2.87	< 0.005
9.26	4.97	< 0.005
5.46	1.08	0.772364
3.33	1.30	0.499231
3.33	1.75	0.093158
2.85	1.64	0.1888
1.90	0.56	0.196667
1.90	0.84	0.703462
1.90	2.54	0.036875
1.66	0.78	0.668667
1.43	0.54	0.244138
0.95	0.62	0.499231
0.71	0.42	0.244138
0.48	1.89	0.505714
0.48	0.52	0.505714
0.48	0.53	0.505714
0.24	1.91	0.668667
0.24	0.15	0.093158
0.24	1.02	0.98
0.24	0.09	< 0.005
0.24	2.57	0.499231
